# Cell-matrix interactions control biliary organoid polarity, architecture, and differentiation

**DOI:** 10.1097/HC9.0000000000000094

**Published:** 2023-03-24

**Authors:** Romina Fiorotto, Valeria Mariotti, Shakila Afroz Taleb, Syeda A. Zehra, Mytien Nguyen, Mariangela Amenduni, Mario Strazzabosco

**Affiliations:** 1 Department of Internal Medicine, Section of Digestive Diseases, Yale School of Medicine, New Haven, Connecticut, USA; 2 Department of Immunobiology, Yale School of Medicine, New Haven, Connecticut, USA

## Abstract

**Background and Aims:**

Cholangiopathies are an important cause of morbidity and mortality. Their pathogenesis and treatment remain unclear in part because of the lack of disease models relevant to humans. Three-dimensional biliary organoids hold great promise; however, the inaccessibility of their apical pole and the presence of extracellular matrix (ECM) limits their application. We hypothesized that signals coming from the extracellular matrix regulate organoids’ 3-dimensional architecture and could be manipulated to generate novel organotypic culture systems.

**Approach and Results:**

Biliary organoids were generated from human livers and grown embedded into Culturex Basement Membrane Extract as spheroids around an internal lumen (EMB). When removed from the EMC, biliary organoids revert their polarity and expose the apical membrane on the outside (AOOs). Functional, immunohistochemical, and transmission electron microscope studies, along with bulk and single-cell transcriptomic, demonstrate that AOOs are less heterogeneous and show increased biliary differentiation and decreased expression of stem cell features. AOOs transport bile acids and have competent tight junctions. When cocultured with liver pathogenic bacteria (*Enterococcus spp.*), AOOs secrete a range of proinflammatory chemokines (ie, MCP1, IL8, CCL20, and IP-10). Transcriptomic analysis and treatment with a beta-1-integrin blocking antibody identified beta-1-integrin signaling as a sensor of the cell-extracellular matrix interaction and a determinant of organoid polarity.

**Conclusions:**

This novel organoid model can be used to study bile transport, interactions with pathobionts, epithelial permeability, cross talk with other liver and immune cell types, and the effect of matrix changes on the biliary epithelium and obtain key insights into the pathobiology of cholangiopathies.

## Graphical abstract

**Figure:**
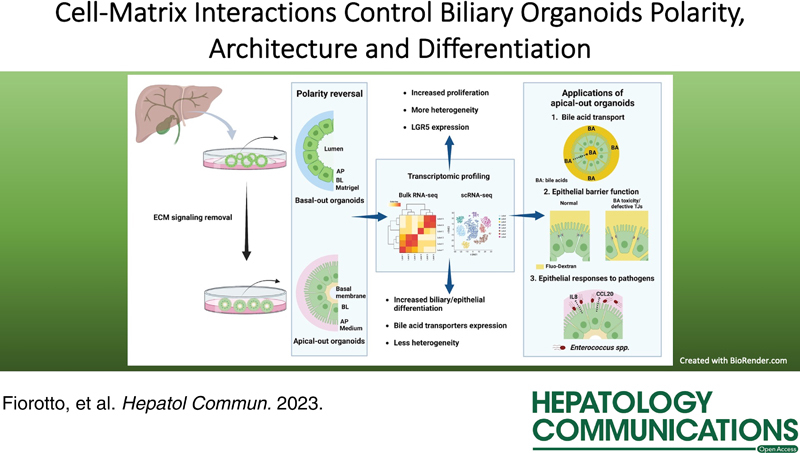

## INTRODUCTION

Cholangiopathies are chronic, progressive, and invalidating diseases that target the biliary epithelium and are still a major unmet need in hepatology, as their etiopathogenesis is unknown and available treatments are unsatisfactory. Reactive/reparatory changes in the biliary epithelium, inflammation, and fibrosis contribute, to a different extent, to the pathogenesis of cholangiopathies, but their mechanisms remain unclear. The lack of human experimental models is a major obstacle to understanding these complex diseases.

The recent availability of tissue-derived 3-dimensional (3D) organotypic cultures provided an advanced tool to understand the relevant pathogenetic mechanisms, discover and validate therapeutic targets, and explore prospects for regenerative medicine.^1^–^4^

Biliary organoids are obtained from freshly isolated liver cells that when cultured on a gel-based extracellular matrix (ECM) [Culturex Basement Membrane Extract (BME) or Matrigel] in a growth factor–defined medium, self-organize in 3D structures enclosing a lumen.^5^ The cells possess morphological and functional features of the biliary epithelium with an apical domain facing the lumen and a basolateral membrane in contact with the matrix. Liver organoids grow indefinitely and can also be pushed toward a hepatocytic-like phenotype, suggesting they preserve a certain degree of plasticity and stemness.^6^

Biliary organoids present important limitations. Accessing the apical domain remains a difficult experimental problem that requires microinjection techniques that can be mechanically disruptive or not commonly available. Furthermore, events and signals coming from the bile (the most relevant for biliary functions) are missed. Also, the study of basolateral events, like the cross talk between cholangiocytes and mesenchymal or immune cells, is complicated by the matrix surrounding the organoid.^7^,^8^

In epithelial cells, the establishment of cell polarity and tissue architecture is tightly regulated by their interactions with the matrix.^9^ The physical properties and biochemical composition of the ECM can influence epithelial cells’ polarity, differentiation, and proliferation.^10^–^12^ For example, stimulation of the integrin receptors at the cell membrane by the ECM informs the orientation of the epithelial cell and initiates the basal membrane deposition (eg, secretion of laminins) and the intracellular signaling cascades responsible for the apical-basal orientation.^13^

We found that the removal of the matrix or inhibition of β-integrin receptor signaling causes an inversion of the apical-basal polarity of biliary organoids that expose the apical membrane. We hypothesized that this change in cell polarity and organoid architecture could enable the study of apical events, such as the transport of bile acids and interactions with microbial pathogens and xenobiotics, and extensively characterized this novel tool to model biliary diseases and study the effect of matrix changes on the biliary epithelium.

## METHODS

Additional methods are reported in the Supplemental Material (http://links.lww.com/HC9/A203) session.

### Human biliary organoids generation and culture

Human biliary organoids were generated from nontransplanted discarded livers or liver explants from patients affected by nonbiliary-related diseases (n=4 livers), as described.^5^,^6^ These deidentified liver tissue samples were obtained from New England Donor Services or through the Yale Liver Center Registry and approved by the Yale University Institutional Review Board (IRB protocol #0603001208) and written informed consent was obtained from all subjects. All research was conducted in accordance with both the Declaration of Helsinki and Istanbul. See Supplemental Material (http://links.lww.com/HC9/A203) for a detailed description and media composition.

### Apical-out organoids generation and culture

Apical-out organoids (AOOs) were generated starting from the organoids embedded in BME as follows. BME-embedded organoids were washed with cold PBS 1X, and the domes were gently resuspended with cold Corning Cell Recovery solution (Corning) and incubated on ice for 1 hour. Matrigel-free organoids were then washed with cold PBS 1X and centrifuged at 4 °C (200*g*, 1 min). The pellet was gently resuspended in expansion media and plated in ultralow attachment 24 multiwell plates (Corning). Media was replaced every other day by spinning down the organoids (200*g*, 1 min).

### Organoid single-cell preparation for 10X genomics

Biliary organoids embedded in BME (EMB), 10% BME, and AAOs were dissociated into single cells. Briefly, domes with EMB organoids were dissolved in ice-cold Corning Cell Recovery solution (Corning) for 10 minutes. After washing with PBS, all organoid types were spun down and resuspended in 0.25% trypsin and incubated for 30 minutes at 37 °C. At the end of the incubation, organoids were mechanically dissociated by pipetting. Trypsin digestion was blocked by the addition of ice-cold resuspension buffer (2% FBS, 1.25 mM CaCl_2_, 4 mM MgCl_2_, 10 mM HEPES, 5 mM glucose in 1X HBSS), and cells were recovered by centrifugation. Cells in the resuspension buffer were filtered through a 35-µm filter (Corning), counted, and checked for viability using Countess II automatic cell counter (Thermo Fisher Scientific). Cells, at the desired concentration, were resuspended in PBS-0.04% BSA.

### Bacteria coculture and imaging

Organoids loaded with CellMask Orange (Thermo Fisher Scientific) were plated on glass-bottom 24-well plates and imaged at ×20 magnification on the stage of a Bruker Opterra II swept-field confocal microscope (Billerica, MA) at 37 °C in a humidified atmosphere containing 5% CO_2_. After the addition of the different bacterial strains, z-stacks (1 μm depth resolution) were acquired every 20 minutes for a total of 16 hours with 488 and 546 nm excitation. Time-lapse files were postprocessed in ImageJ (NIH, Bethesda, MD).

### Statistical analysis

Data other than sequencing data were analyzed and plotted using GraphPad Prism (Version 9.2.0) software. Data were presented as mean±SD. Differences between 2 groups were assessed for statistical significance using the 2-tailed unpaired *t* test. ∗∗ indicates *p*<0.01; ∗ indicates *p*<0.05. When performing multiple comparisons, significance was assessed by ANOVA, followed by Sidak or Kruskal-Wallis multiple comparison tests.

### Data availability

The bulk and scRNA data generated in this study have been deposited in GEO Repository under the accession code GSE204960.

## RESULTS

### Liver organoids reverse their apicobasal polarity when removed from ECM

**FIGURE 1:**
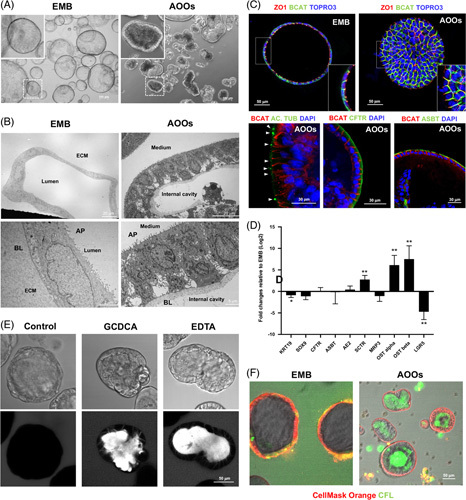
In the absence of BME, biliary organoids switch their apical-basal polarity. (A) Brightfield images comparing biliary organoids grown embedded in BME (EMB) or in suspension (AOOs) at day 7 from removal from the ECM. AOOs show a change of morphology with a lack of the typical spheroidal shape. Scale bars are indicated. (B) Representative EM images of EMB organoids and AOOs showing the opposite orientation of the apical and basolateral membrane. In EMB, the apical surface covered by microvilli is oriented toward the internal lumen, whereas the basolateral membrane is in contact with the ECM. Differently, AOOs are characterized by a more columnar epithelium with basolateral nuclei and an outward apical membrane in contact with the medium. The basolateral membrane is in contact with an internal cavity. Scale bars 2, 5, 20 μm. (C) Confocal images of EMB and AOO organoids costained for β-catenin (ie, basolateral marker), ZO-1 (ie, apical marker), and Topro3 (ie, nuclear staining). In organoids embedded into the ECM, ZO-1 marks the cell-cell contacts on the internal luminal side while β-catenin is distributed along the basolateral membrane in contact with the ECM. On the other hand, in AOOs, the expression of both markers was reversed, with ZO-1 expressed toward the side facing the medium and β-catenin toward the inside, confirming the polarity switch in the absence of ECM. AOOs were also costained for β-catenin/acetylated α-tubulin (ie, cilium marker)/DAPI, β-catenin/CFTR/DAPI, and β catenin/ASBT/DAPI that showed the presence of apical cilia oriented toward the external surface (see arrows) and the apical expression of CFTR and ASBT. (D) qRT-PCR analysis to compare changes in mRNA expression of biliary markers in AOOs versus EMB; mean ± SD organoids derived from n=4 livers; *p<0.05, **p<0.01. € Transmitted light and confocal images of FITC fluorescent dextran assay to measure epithelial barrier integrity in AOOs control or after treatment with GCDCA (500 mM) or EDTA (2 mM). Paracellular diffusion of fluorescent dextran is shown after the treatment with GCDCA and EDTA. (F) Bile acid transport assay in EMB and AOOs. Merged transmitted light and confocal images of organoids stained with CellMask Orange (red) to mark the plasma membrane and preincubated with the fluorescent bile acid cholyl-lysyl-fluorescein, CLF (green). Bile acid fluorescence is shown inside the cells but not in the lumen in EMB, contrary to AOOs, where the bile acid is accumulated in the internal cavity. Images are representative of n=3 independent experiments. Abbreviations: AOOs, apical-out organoids; AP, apical membrane; BL, basolateral membrane; ECM, extracellular matrix; EMB, organoids embedded in BME.

We generated biliary organoids as described^5^,^6^ and cultured them in BME as 3D spheroids with an enclosed lumen. Here, we will call this classic organoid culture EMB (from embedded into BME). EMB expresses the expected biliary markers such as *KRT19*, *SOX9*, *CFTR*, *SLC4A2*, and the stem cell marker *LGR5* 5,14 (Figure 1D and Supplemental Figure 3, http://links.lww.com/HC9/A194).

When removed from the BME (see Methods), without disrupting their integrity, and placed into a floating culture, EMB organoids changed in morphology and acquired a less spheroid shape with a thick cuboidal monolayer of cells surrounding one or more smaller cavities. These changes started as early as day 1, and by day 7, all the EMB organoids had changed their 3D architecture (Figure 1A). On the contrary, no change in morphology was evident when organoids were cultured in a fluid suspension of medium containing only 10% of BME (Supplemental Figure 1, http://links.lww.com/HC9/A192).

Transmission electron microscope analysis showed that in the absence of BME, the surface covered by distinct microvilli (the apical domain) had an opposite location compared with the EMB organoid (Figure 1B and Supplemental Figure 2A, B, http://links.lww.com/HC9/A193). Indeed, the apical surface was oriented toward the external medium rather than facing the enclosed lumen of the 3D structure as in EMB organoids (Figure 1B), indicating the reversal of cell polarity. Apical-out organoids (thereafter named AOOs) showed a more columnar epithelium characterized by a basolateral nucleus (Figure 1B and Supplemental Figure 2A, B, http://links.lww.com/HC9/A193), well-preserved tight junctions and many mitochondria and secretory vesicles.

To confirm the polarity switch in AOOs, we performed immunofluorescence and confocal imaging analysis for proteins that specifically define the apical or basolateral membrane in polarized cells, such as ZO-1 to mark tight junctions in the apical domain and β-catenin to delineate the basolateral domain^15^ (Figure 1C). In organoids embedded into the ECM, ZO-1 marks the cell-cell contacts on the internal luminal side while β-catenin is distributed along the basolateral membrane in contact with the ECM. On the other hand, in AOOs, the expression of both markers was reversed, with ZO-1 expressed toward the side facing the medium and β-catenin toward the inside (Figure 1C), confirming the polarity switch in the absence of ECM. Moreover, immunofluorescence for acetylated α-tubulin confirmed in AOOs the presence of primary cilia oriented toward the external surface (Figure 1C).

To understand how the rearrangement of polarity takes place in AOOs, we performed time-lapse experiments. The plasma membrane was stained with the fluorescent vital dye CellMask Orange, and then organoids were imaged for several hours after the removal of the ECM. As shown in Supplemental Video 1, the organoid folded and underwent a process of invagination that resulted in an inside-out eversion. Processes such as folding and invagination are common during the development and seem to be mediated by β-integrin-RhoA-myosinII^16^,^17^ and initiated by a stressor that, in our case, could be the removal of the matrix.

**Video 1:** 

### AOOs have a well-defined biliary profile

#### Biliary markers

To investigate the phenotypic profile of AOOs, we first determined the gene expression of biliary markers by RT-PCR and compared it with EMB. As shown in Figure 1D and Supplemental Figure 3, (http://links.lww.com/HC9/A194), gene expression of *KRT19*, *SOX9*, *CFTR*, *SLC4A2* (ie, AE2), *SLC10A2* (ie, ASBT), and *MRP3* in AOOs was similar to that of organoids embedded in BME. However, genes related to bile acids transport, such as *SLC51A/B* (ie, OSTα/β),^18^ and to biliary secretion, such as the secretin receptor,^19^ were strongly upregulated in AOOs, whereas the expression of the stem cell marker *LGR5* was almost undetectable. The apical localization of CFTR and ASBT was confirmed by immunofluorescence (Figure 1C). These data suggest the acquisition of a more mature biliary phenotype in AOOs.

#### Bile acids transport

The biliary epithelium is constantly exposed to high concentrations of bile acids from the apical side. Bile acids can act as signaling molecules when binding to the nuclear farnesoid X receptor and the membrane G protein–coupled bile acid receptor 1 (TGR5).^20^,^21^ Furthermore, cholangiocytes can transport bile acids from the bile to the circulation or “cholehepatic shunt.”^22^ As organoids express bile acid transporters transcripts, we assessed whether they have the capability to vectorially transport bile acids using the fluorescent bile acid derivative cholyl-lys-fluorescein. Both embedded organoids and AOOs were incubated with cholyl-lys-fluorescein and then imaged with a confocal microscope (Figure 1F). As shown in Figure 1F, in embedded organoids, cholyl-lys-fluorescein enters the cells but cannot be excreted into the lumen. On the contrary, AOOs were able to internalize the bile acid from the apical side and excrete it into the internal cavity confirming that AOOs, but not EMB, have the capability to vectorially transport bile acids and mimic their physiological transport from the bile to the bloodstream.

#### Paracellular permeability

Although allowing the regulated transport of bile acids, the biliary epithelium also establishes a physical barrier to protect the surrounding tissues from their high concentrations in the lumen. In normal conditions, the barrier function maintained by competent tight junctions^23^ can be altered in inflammation or in case of exposure to toxic bile acids, as is the case in chronic cholestasis.^24^ We assessed the epithelial barrier function in AOOs by exposing them to 4 kDa FITC-dextran, followed by confocal imaging. Figure 1E shows that AOOs are not permeable to the fluorescent dextran, therefore, confirming the integrity of the epithelial barrier. However, preincubation with glycochenodeoxycholic acid (500 μM), a toxic bile acid present in high concentration in the bile of cholestatic patients,^25^ increased the permeability to the dextran that diffused paracellularly into the internal lumen (Figure 1E). A similar effect was also seen in AOOs pretreated with EDTA, a calcium chelator known to disrupt the tight junction structure (Figure 1E).

### Transcriptomic analysis indicates improved epithelial maturation in AOOs

**FIGURE 2:**
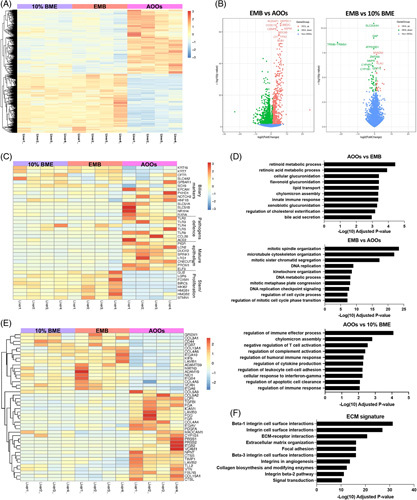
Bulk RNA-Seq analysis indicates an improved epithelial maturation in AOOs and the perturbations of β1 integrin signaling between EMB and AOOs. Biliary organoids (derived from n=4 human livers) were cultured embedded in BME (EMB), in suspension in a 10% BME/medium mix (10% BME) and in suspension in the medium without ECM (AOOs) and processed for bulk RNA-sequencing. (A) Heatmap showing differentially expressed genes (DEGs) in organoids generated from 4 different livers and cultured as EMB, 10% BME, and AOOs based on *p* adj<0.05 and FC cutoff=2. (B) Volcano plots showing DEGs that are upregulated or downregulated in EMB versus AOOs (2282) and EMB versus 10% BME 26 based on *p* adj<0.05 and FC cutoff=2. (C) Heatmap showing DEGs of selected genes related to biliary differentiation markers, pathogens defense-related genes, mature epithelium markers, stemness, and proliferation markers. (D) Gene Ontology analysis of differentially expressed genes between AOOs versus EMB, EMB versus AOOs, and AOOs versus 10% BME, respectively, using EnrichR, listing terms identified with bars indicating the level of significance (adjusted *p*-values). (E)Heatmap showing DEGs of selected genes related to an extracellular matrix organization. (F) Pathway enrichment analysis of DEGs related to extracellular matrix organization in EMB versus AOOs indicates the perturbations of β-integrin signaling in response to ECM removal (BioPlanet 2019). Abbreviations: AOOs, apical-out organoids; BME, Basement Membrane Extract; ECM, extracellular matrix; EMB, organoids embedded in BME.

To better understand the transcriptomic changes induced in biliary organoids by manipulating the ECM, we performed bulk RNA-sequencing in biliary organoids obtained from 4 individual livers cultured as EMB or in a fluid suspension of medium containing 10% v/v of BME (10% BME) or as AOOs. We first performed a principal component analysis of the top 200 variable genes among the different conditions. As shown in the heatmap and the PCoA plot in Figure 2A and Supplemental Figure 4 (http://links.lww.com/HC9/A195), organoids cultured in the presence of ECM (EMB and 10% BME) share a very similar transcriptomic profile. In contrast, a larger variation is present between organoids cultured in the presence of ECM (EMB and 10% BME) and AOOs. Differential gene expression (DEGs) analysis (Supplemental File 1, http://links.lww.com/HC9/A199) showed that over 2000 genes were differentially expressed between AOOs and EMB (2282 genes) or 10% BME (2054 genes), whereas only 43 genes were differentially expressed between EMB and 10% BME (Figure 2B). At first, we examined the expression pattern of selected genes specific for a biliary signature (Figure 2C). *KRT19*, *KRT7*, and other specific functional biliary markers such as *CFTR*, *SLC4A2*, *SOX9*, *EPCAM*, and *GPBAR1* were similarly expressed. Of note, the expression of cholangiocyte-specific bile acid transporters, such as *SLC51A* (OSTα) and *SLC51B* (OSTβ), as well as nuclear receptors *NR1H4* and *RXRA*, was significantly increased in AOOs, confirming their ability to transport and respond to bile acid signaling. The expression of genes involved in epithelial cell response to PAMPs and DAMPs, such as *TLRs (2-3-9)*, *CCL20*, and *ACE2*, the host receptor for SARS-CoV-2 entry, was higher in AOOs. Also, several genes involved in epithelial differentiation or expressed by mature biliary cells in the liver^27^ (ie, *PIGR*, *LCN2*, *DUOX2*, *LRG1*, *ONECUT2*, *PROM1*, and *ELF3*) were upregulated in AOOs, whereas the stem cell marker *LGR5* and the mesenchymal marker *GLI2* were significantly downregulated. Several genes associated with cell proliferation (ie, *FOXM1*, *BIRC5*, *MKI67*, and *HMGB1/2*) were also downregulated in AOOs. A similar pattern of differential expression toward a less proliferative but more mature biliary/epithelial phenotype by AOOs was further confirmed by comparison with a public data set (GSE146899) (Supplemental File 2, http://links.lww.com/HC9/A200) produced using primary normal human cholangiocytes lines in monolayer^28^ (Supplemental Figure 5, http://links.lww.com/HC9/A196).

To better understand these differences, we performed Gene Ontology (GO) analysis of the top 200 DEGs (Figure 2D). Genes upregulated in EMB and 10% BME organoids were enriched for GO terms related to cell cycle and cell division, suggesting a more proliferative profile phenotype compared with AOOs, and further confirmed by EdU staining (Supplemental Figure 6, http://links.lww.com/HC9/A197).

Genes upregulated in AOOs compared with EMB were enriched for GO terms involved in metabolic processes, lipid transport, innate immune responses, and bile acid secretion. Because of a decreased cell proliferation, we searched for GO terms related to cell senescence, but such a signature was not present among the significant (adjusted *p* value <0.05) biological processes (Supplemental File 3, http://links.lww.com/HC9/A201). Interestingly, genes upregulated in AOOs compared with 10% BME organoids were enriched in GO terms related to the regulation of immune response, complement activation, and cytokines production. We also identified a group of 23 DEGs involved in cell-matrix adhesion (GO: 0007160) between EMB and AOOs (Figure 2E). Genes upregulated in AOOs are related to ECM-associated components, such as collagens (*COL5A3*, *COL9A2*, and *COL16A1*), laminins (*LAMB2* and *LAMB3*), ECM homeostasis (*FGA*, *FGG*, *FGB*, and *VTN*), growth factors (*TGFβ1* and *PDGFA*), and integrins (*ITGAV* and *ITGB2*). EnrichR analysis of these 23 genes showed they were related to β1integrin signaling (Figure 2F).

**FIGURE 3:**
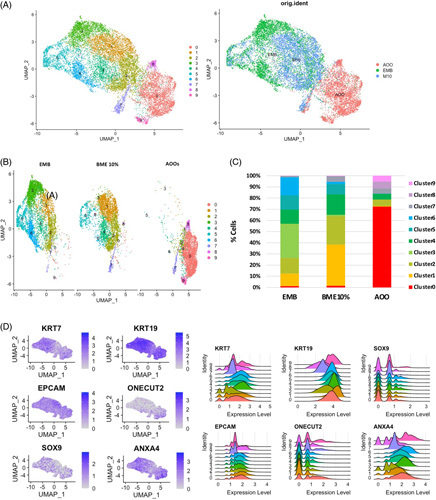
scRNA-seq analysis shows that expression of biliary features is present in all 3 types of organoids and reveals decreased transcriptional heterogeneity in AOOs. (A) UMAP plots showing clustering of 13,700 analyzed cells from EMB, 10% BME, and AOOs organoids and their original identity. (B) Uniform Manifold Approximation and Projection (UMAP) plot showing the distribution of identified clusters in the 3 different types of organoids (EMB, 10% BME, and AOO). (C) Percentage of cells per cluster in each type of organoids, showing a higher heterogeneity in EMB and 10% BME compared with AOOs. (D) Feature plots and ridge plots showing the distribution of biliary markers across all clusters of cells from the different organoids. Abbreviations: AOOs, apical-out organoids; BME, Basement Membrane Extract; EMB, organoids embedded in BME; UMAP, Uniform Manifold Approximation and Projection.

We then performed scRNA sequencing in EMB, 10% BME, and AOO organoids. Unsupervised clustering analysis identified a total of 10 clusters (0–9), with a different distribution within the 3 conditions (Figure 3A–C and Supplemental File 4, http://links.lww.com/HC9/A202). EMB organoids seemed to be more heterogeneous, with cells equally distributed among clusters 1 (11.25%), 2 (13.99%), 3 (30.58%), 4 (12.56%), 5 (12.87%), and 6 (16.18%). Similarly, in 10% BME organoids, most cells were distributed among clusters 1 (36.81%), 2 (25.31%), 4 (18.36%), 5 (9.24%), and 7 (5.01%). Conversely, AOOs showed less heterogeneity, with the majority of cells belonging to cluster 0 (72.32%) and the rest distributed among clusters 2 (5.64%), 4 (5.21%), 7 (4.68%), 8 (7.09%), and 9 (5.31%) (Figure 3C).

**FIGURE 4:**
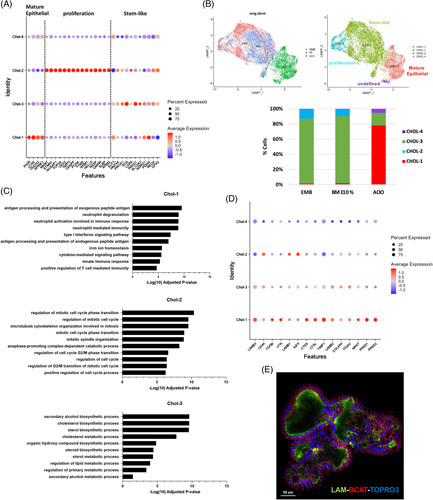
scRNA-seq analysis identifies 3 main populations of cholangiocytes among the different types of organoids. (A) The dot plot shows the expression of specific genes used by supervised analysis to identify different populations of cholangiocytes. The size of the circle is proportional to the percentage of cells expressing each gene, whereas the intensity of the color indicates the average expression level. (B) UMAP plots showing the distribution of the identified populations of cholangiocytes among the 3 different types of organoids compared with their original identity. The bar graph shows the percentage of the cell populations in each type of organoid. (C) Gene Ontology analysis of differentially expressed genes in Chol-1, Chol-2, and Chol-3 using EnrichR. A list of terms is identified with bars indicating the level of significance (adjusted *p*-values). (D) The dot plot shows the changes in the expression of selected genes related to extracellular matrix organization in the different populations of cholangiocytes. The size and color of the circles are as explained in A. (E) Confocal image of AOOs organoids costained for laminin [extracellular matrix (ECM) component marker], β-catenin (ie, basolateral marker), and Topro3 (ie, nuclear staining) showing the presence of ECM on the internal cavity of the organoid. Scale bar 50 mm. Abbreviations: AOOs, apical-out organoids; BME, Basement Membrane Extract; EMB, organoids embedded in BME; UMAP, Uniform Manifold Approximation and Projection.

All the clusters expressed biliary markers such as *KRT7*, *KRT19*, *SOX9*, *EPCAM*, *HNF1b*, and *ANXA4* suggesting that all the 3 types of organoids retain the transcriptomic profile of cholangiocytes (Figure 3D). Using a recently published data set,^27^ we performed a supervised analysis on DEGs and grouped the different cell clusters in 4 main populations of cholangiocytes (Chol-1, Chol-2, Chol-3, and Chol-4) (Figure 4A, B, Supplemental Figure 7, http://links.lww.com/HC9/A198 and Supplemental File 4, http://links.lww.com/HC9/A202). Chol-1 included clusters 0 and 8 and were marked by a higher expression of *PIGR*, *LCN2*, *DUOX2*, *SPINK1*, and *ELF3* that are expressed by mature epithelia (Figure 4A and Supplemental Figure 7, http://links.lww.com/HC9/A198). By DEGs analysis, Chol-1 showed enrichment in biological processes related to antigens processing and presentation and pathogens defense (Figure 4C). Chol-2 was uniquely represented by cluster 5 that identified cholangiocytes in a proliferating state as shown by the high expression of cell cycle-related genes such as *MKI67*, *PCNA*, *CKS2*, and *NASP* (Figure 4A, B and Supplemental Figure 7, http://links.lww.com/HC9/A198) and further confirmed by gene enrichment analysis (Figure 4C). Chol-3 (ie, clusters 1, 2, 3, 4, 6, and 7) identified a population of cholangiocytes also expressing genes typical of hepatocytes (ie, *HNF4A*, *PROX1*, *SCD*, *INSIG1*, *SLC2A3*, *NDRG1*, and *PDIA4*) and of intestinal cells (ie, *REG4* and *GPX2*) (Figure 4A and Supplemental Figure 7, http://links.lww.com/HC9/A198). DEG analysis of Chol-3 showed enrichment in biological processes related to alcohol and lipid metabolism (Figure 4C). Finally, Chol-4 included mainly the cells belonging to cluster 9 and showed no distinctive feature (Figure 4A and Supplemental Figure 7, http://links.lww.com/HC9/A198).

Feature plots in Figure 4B show the distribution of the 4 populations of cholangiocytes among the 3 types of organoids. It is evident that AOOs are mainly composed of Chol-1 with a more mature epithelial phenotype, whereas EMB and 10% BME are homogeneously represented by Chol-2 and Chol-3 that include high-proliferating cells and cholangiocytes coexpressing other cell type features. Interestingly, many cells in Chol-2 and 3 also expressed the stem cell marker *LGR5*, suggesting a more transitional/stem-like phenotype (Figure 4A). In contrast, none of the cells isolated from AOOs were represented by Chol-2 (Figure 4A), confirming a reduced level of proliferation in AOOs as reported above.

Finally, we searched, in our single-cell data set, the genes involved in cell-matrix adhesion previously identified by the bulk RNA-seq analysis. Although some genes were not captured by scRNA-seq, most of them were upregulated in Chol-1 (Figure 4D), suggesting a process of matrix deposition/rearrangement activated in the absence of ECM. To corroborate this finding, we stained AOOs for laminin, a main component of the basal membrane that is known to be secreted during the establishment of apical-basal cell polarity.^13^ As shown in Figure 4E and Supplemental Video 2, we confirmed the presence of laminin in contact with the basolateral membrane in the internal cavity of AOOs, suggesting a *de novo* secretion of the basal membrane after the removal of the ECM.

**Video 2:** 

### Interaction of β1 integrins with ECM determines the polarity orientation of liver organoids

On the basis of the RNA-seq data, showing a divergent expression of genes related to β1 integrin pathway between EMB organoids and AOOs, we investigated whether the specific inhibition of the β-integrins/ECM interaction influenced the organoid polarity. Both EMB and 10% BME organoids were exposed to a specific blocking antibody for β1 integrin (ABII2) and observed in culture for 4 days.

**FIGURE 5:**
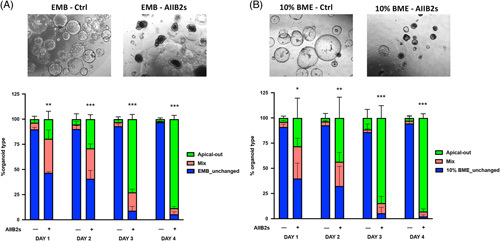
Blockage of β1 integrin signaling induces apical-out polarity in biliary organoids cultured in BME. Organoids were cultured as EMB (A) and 10% BME (B) and were treated with an anti-β1 integrin–neutralizing (ABII2s) antibody diluted to 2.2 μg/mL in Expansion Media for 4 days. Changes in polarity (apical-out) were determined based on the organoid morphology. Brightfield images of random fields/wells were acquired with an Olympus CKX41 from day 1 to day 4. Organoids were classified as classic (ie, spheric with a clear lumen delimited by a thin layer of cells), apical out (ie, presence of a cavity delimited by a thick epithelium), or mix (ie, partial apical out and partial classical or organoids in reversal phase with no cavity visible) based on their morphology and were manually counted from independent wells (n=3 wells for each experimental condition for a total of n=4 organoid lines). The number of organoids of each type was calculated as a percentage of the total number of organoids. Untreated organoids served as controls. Representative transmitted light images show EMB (A) and 10% BME (B) organoids on day 4 for control or after treatment. Bars show the percentage of organoids in the different configurations (ie, apical-out, mixed morphology, EMB unchanged, or 10% BME unchanged), based on their morphology for each of the 4 days (means ± SD from n=4 organoid lines, each analyzed in triplicate; 1-way ANOVA, followed by Sidak multiple comparison test was performed for selected comparisons (ie, treated versus untreated for each day and untreated among them for each day, ***p<0.001, **p<0.01, and *p<0.05, respectively. Abbreviations: BME, Basement Membrane Extract; EMB, organoids embedded in BME.

As shown in Figure 5, treatment with the blocking antibody progressively changed the morphology of both EMB and 10% BME organoids into that of AOOs, consistent with a switch of polarity. Quantification of these changes revealed that at day 4, up to 88.5% of EMB (Figure 5A) and 92.9% of 10% BME organoids (Figure 5B) assumed an apical-out configuration, whereas no changes were present in the respective untreated controls. These data further suggest that changes in the ECM/integrin binding, rather than changes in ECM composition/stiffness, are responsible for the apical-out polarity in AOOs.

### AOOs allow the study of host-microbe interaction at the epithelial surface

**FIGURE 6:**
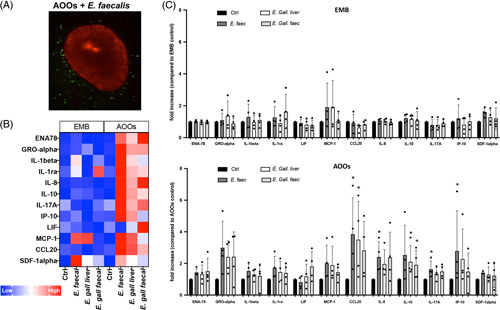
AOOs secrete chemokines when cocultured with heat-inactivated bacteria. (A) Representative confocal image showing live AOOs labeled with CellMask Orange (in red) in coculture with live bacteria strain *E. faecalis* labeled with CSFE (in green). See also Supplemental Video 3. EMB and AOOs were cocultured with heat-inactivated *Enterococcus spp.* (*E. faecalis*, *E. Gallinarum liver*, and *E. Gallinarum faecalis*) for 6 hours, and the supernatant was collected to measure the secretion of chemokine and cytokines by the proteomic array. After processing, spot pixel density was measured in automatic using image analysis software (see Methods section for details). (B) The heat map depicts average expression changes in selected inflammatory mediators secreted in the media by organoids. (C) Columns represent the average fold increases in protein expression normalized to control organoids (not exposed to bacteria). Data are expressed as means ± SD, in n=4 organoid lines and 1-way ANOVA, followed by Kruskal-Wallis multiple comparison tests performed to compare each bacteria coculture versus controls, *p<0.05). Significant changes in the secretion of inflammatory mediators were evident in AOOs but not EMB organoids. Abbreviations: BME, Basement Membrane Extract; EMB, organoids embedded in BME

An important function of epithelia is to protect the host against pathogens and pathobionts invasion by creating a physical barrier at the interface and by communicating with the immune system for prompt intervention. The biliary epithelium is exposed to pathobionts coming from the intestine and the bile, and bacterial infections are a common feature in cholangiopathies.^29^ As the apical surface is not accessible in embedded organoids, whereas in AOOs, the apical domain is easily accessible, we tested their response to members of the *Enteroccoccus* bacterial family, known pathobionts of the liver and biliary tract.^30^–^32^ We cocultured AOOs with *Enterococcus ssp.* and live imaged them for 8 hours. As shown in Figure 6A and Supplemental Video 3, bacteria established physical contact with the epithelial cells but were not able to colonize the organoids. To study whether interaction with bacterial components elicits a proinflammatory response in the biliary epithelium, we cultured both EMB and AOOs with heath inactivated (to avoid confounding effects due to their growth in the media) *Enteroccoccus faecalis, Enteroccoccus gallinarum liver*, and *Enteroccoccus gallinarum faecalis*. After 6 hours of coculture, we measured the presence of chemokines and cytokines in the supernatant using a proteomic array. As shown in Figure 6B, all bacteria species, but to a different extent, were able to stimulate the secretion of chemokines and cytokines typical of epithelial innate immune responses after exposure to PAMPs, such as ENA-78, IL1β, IL8, IP-10, CCL20, IL17α, and SDF1α in AOOs but not in EMB organoids. Notably, CCL20, IL8, IL17 α, and IP-10 were significantly upregulated in response to *E. faecalis*, and CCL20 was 3–4 times more elevated compared with controls in all the bacteria species (Figure 6C). These data are consistent with previous findings showing the secretion of CCL20 by biliary cells in response to LPS.^33^ In fact, CCL20 is a well-known chemoattractant of T lymphocytes and neutrophils and was recently shown to be involved in the cross talk between biliary epithelial cells and monocytes in response to pathogens and to promote a Th17-polarizing microenvironment in inflammatory biliary diseases as PSC and PBC.^33^,^34^

**Video 3:** 

The absence of a response in EMB organoids could be caused by the presence of the BME matrix that limits the contact of the organoids with the PAMPs.

## DISCUSSION

Biliary organoids are increasingly used to model biliary diseases.^1^,^2^,^4^ Organoids are embedded into BME/Matrigel matrix, a complex laminin-rich basement membrane matrix extracted from the Engelbreth-Holm Swarm mouse sarcoma that provides the isolated stem/progenitor cells, the necessary support to self-assemble into 3D structures and differentiate.^35^ The biological complexity and physical limitations of these matrices, however, represent a challenge for many experimental applications.

Given the influence of the ECM on epithelial tissue differentiation and architecture, we decided to study the effects of manipulating the ECM on biliary organoids that were previously cultured inside the BME/Matrigel. Our results show that when the ECM was removed, organoids underwent a rapid architectural change that resulted in a complete inversion of the apical-basal polarity while still maintaining a 3D conformation. This rearrangement occurs within hours and involves a dynamic folding and invagination process (Supplemental Video 1), as proposed for human enteroids.^36^ The switch of polarity was confirmed by immunolocalization of specific polarity markers and by transmission electron microscope analysis. Notably, exposure of organoids to diluted ECM (10% BME) was enough to maintain the organoid in their original configuration and prevent the change in polarity.

Regulation of epithelial cell polarization requires a coordinated series of events that begin when the cell establishes physical contact with the ECM.^9^ Among them are the interactions mediated by integrin receptors (mainly α2β1 heterodimers) that bind collagens and laminins. The site of interaction between cell adhesion integrin receptors and the ECM defines the basal cell domain.^13^ This initial step is followed by the organization of the cytoskeleton that will regulate the transport of specific proteins to the apical plasma membrane. Before that step, apical and basolateral proteins are asymmetrically distributed on the plasma membrane.^13^ Using transcriptome profiling, we performed a supervised analysis to search for genes involved in ECM remodeling and cross talk. By enrichment analysis, we identified a set of genes upregulated in AOOs that play a role in the process of cell adhesion to the ECM and are related to β1integrin receptor signaling.

By treatment of biliary organoids, embedded in the ECM or 10% BME, with an antibody that blocks β1 integrin-ECM interaction we confirmed the role of β1 integrin in the process. Indeed, the organoids inverted their polarity and assumed an apical-out configuration.

It is unclear why the removal of ECM determines a reorientation instead of causing a complete loss of polarity. Perhaps, the removal of ECM causes mechanical stress to the organoid that starts contracting and folding and possibly secreting a new basal membrane. Indeed, we confirmed that, from the basolateral side, AOOs are in contact with a basal membrane containing laminin; this was likely secreted in response to the loss of polarity in the presence of cell-cell contacts (Figure 4E).

The interaction with the ECM is essential during the development of the biliary epithelium,^37^ raising the question of whether the differentiation of the organoid was affected by ECM removal. On the contrary, our transcriptomic data demonstrate that after the removal of the ECM, AOOs maintain their biliary profile, express the established biliary markers, and are more differentiated. Because in our experimental conditions, the ECM is removed after organoids are formed, it is likely that their biliary commitment is maintained by the growth factors contained in the media. Moreover, we speculate that chemical and physical signals coming from the apical side, together with the basolateral secretion of a basal membrane of laminin,^38^ are promoting their full biliary differentiation. Indeed, bulk RNA-seq data showed that AOOs have higher expression levels of bile acid transporters (ie, *OSTα* and *OSTβ*)^18^ and nuclear receptors (ie, *NR1H4* and *RXR*)^21^ as well as genes typical of differentiated epithelia (ie, *PIGR*, *PROM1*, and *ELF3*) whereas the expression of the stem cell marker *LGR5* becomes almost undetectable. ScRNA-seq data confirmed that AOOs are the homogeneous population of cholangiocytes expressing mature epithelial markers. On the other hand, organoids cultured in the presence of BME (both EMB and 10% BME) have more stem-like properties, as suggested by the expression of *LRG5* and of a few genes present in hepatocellular and intestinal transcriptomes. Furthermore, with respect to AOO, EMB organoids contain a cell population highly enriched in genes associated with cell cycle and proliferation, therefore, confirming their self-renewal capability, as described.^6^ Altogether, these findings suggest that AOOs have a more mature biliary phenotype compared with organoids embedded in the ECM; perhaps growth factors present in the BME maintain a local microenvironment that support the stem cell niche.

Of note, in 10% BME organoids, there was no change in polarity, and their transcriptomic profile was very similar to BME organoids, suggesting that mechanical forces exerted by the ECM have a lower impact on the polarity orientation and differentiation in biliary organoids. The advantage of the 10% BME model is that the organoids are in a less dense environment compared with EMB and, therefore, ideal to study basolateral interactions with other cells, such as mesenchymal and immune cells.

The luminal uptake of certain bile acids by cholangiocytes and their secretion on the basolateral side allows the local recycling of bile acids from the bile and back into the hepatocyte (cholehepatic shunting).^22^ Consistent with their differentiated transcriptome, AOOs were able to uptake apically a fluorescent bile acid and vectorially transport it out through the basolateral membrane. On the contrary, when EMB are exposed to the same fluorescent bile acid, this is uptaken by the basolateral membrane but not secreted into the lumen. Thus, AOOs will prove to be an excellent model to study biliary secretory and absorptive functions and to test toxicants and therapeutic molecules, experiments that otherwise would not be possible in the classic conformation inside the BME/Matrigel.

We observed that AOOs maintain a well-preserved barrier function. This is based on the electron microscopy analysis, showing the presence of TJs sealing the paracellular space, and on the permeability assay showing the exclusion of 4 kDa dextran. After exposure to high concentrations of the toxic bile acid glycochenodeoxycholic acid, we observed the paracellular transit of the fluorescent dextran from the apical to the basolateral compartment, suggesting a loss of TJs integrity. Alteration of TJs and associated biliary dysfunction have been described in the pathogenesis of several cholestatic diseases, among these, PBC, PSC,^39^,^40^ and biliary atresia^1^ and in rare genetic cholangiopathies such as cystic fibrosis^15^ and PFIC4.^41^ The availability of apical-out biliary organoids derived from patients and the possibility of studying defects in their barrier function in response to bile acids, xenobiotics, and infectious agents will have a great impact.

The biliary epithelium acts as a first line of defense against bacteria, which are often found in normal and pathological bile.^42^,^43^ Given the increasing role assigned to the liver-gut axis in several liver and biliary diseases,^26^ we have explored the possibility of establishing a model to study the interaction between bacteria and epithelial cells using AOOs. Transcriptomic analysis showed that genes related to pathogen defense are upregulated in AOOs, as seen in mature epithelial cells. Therefore, we devised an *in vitro* coculture system in which we used known biliary pathobionts belonging to the *Enterococcus spp* (ie, *E. faecali*s, *E. gallinarum liver*, and *E. gallinarum faecalis*) that translocate from the gut to the liver.^31^,^32^ The bacteria were not able to pass through the epithelial barrier of AOOs but triggered the secretion of inflammatory mediators typical of the epithelial response to pathogens, such as IL8, IP-10, and CCL20. Notably, CCL20 is known to be secreted by the biliary epithelium in response to pathogens and has been implicated as a trigger of the chronic Th17 response in a patient with primary sclerosing cholangitis (PSC) and in PSC experimental models.^44^,^45^ On the contrary, EMB organoids exposed to the same bacteria lack a response by not showing secretion of inflammatory mediators. Thus, these data validate the importance of using our in vitro coculture system to study the response of the biliary epithelium to gut-derived pathobionts.^26^

In conclusion, the interaction with the ECM has significant effects both on the structure and the functional maturation of the epithelium. By modulating these interactions and inverting the polarity of the epithelium, one can significantly increase the range of applications of organoid models to study biliary diseases. In fact, apical-out organoids recapitulate important functions of the biliary epithelium and allow easy access to the apical membrane, therefore, expanding potential applications. Further studies comparing the effect of different types of matrices will give important clues on the cross talk between the epithelium and the ECM.

## Supplementary Material
