# Supplementary figures and images for: Cell-matrix interactions control biliary organoid polarity, architecture, and differentiation
Source: Hepatol Commun. 2023 Mar 24;7(4):e0094. doi: 10.1097/HC9.0000000000000094 (PMC10503667; doi:10.1097/HC9.0000000000000094)

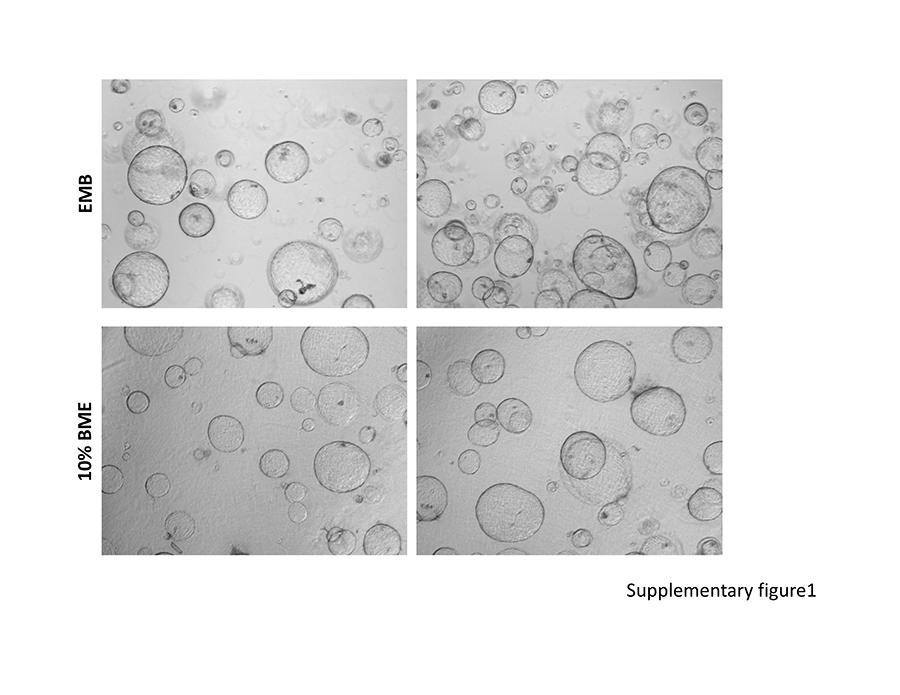

Supplement: Supplementary file 4 [file hc9-7-e0094-s001.tif]

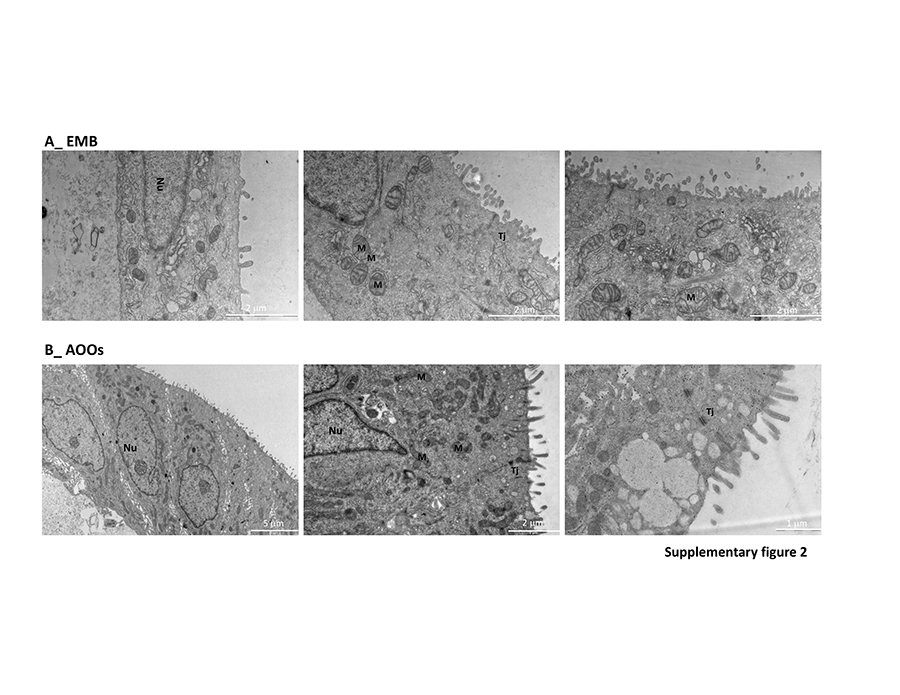

Supplement: Supplementary file 5 [file hc9-7-e0094-s002.tif]

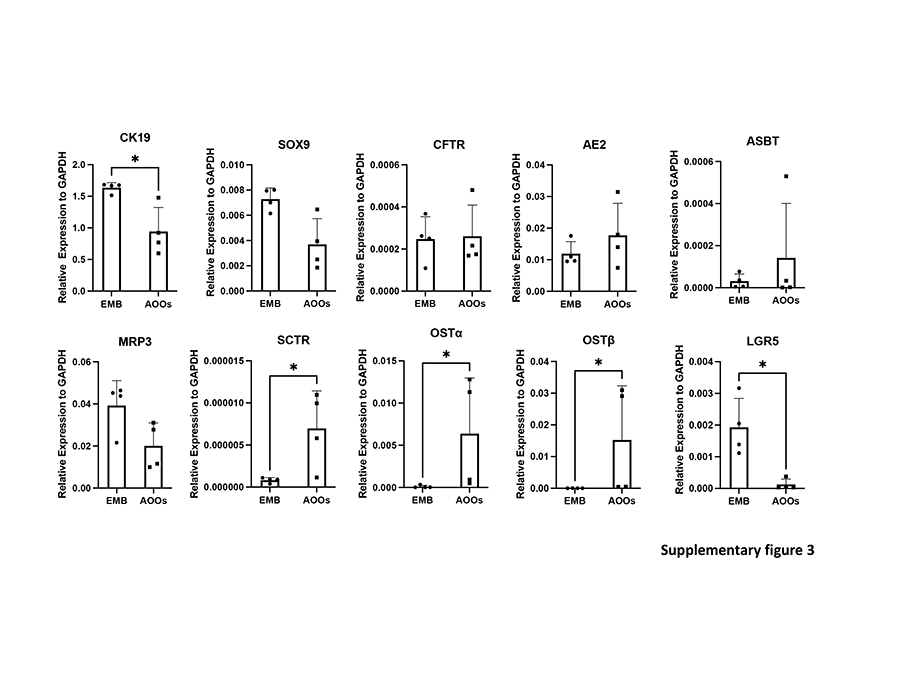

Supplement: Supplementary file 6 [file hc9-7-e0094-s003.tif]

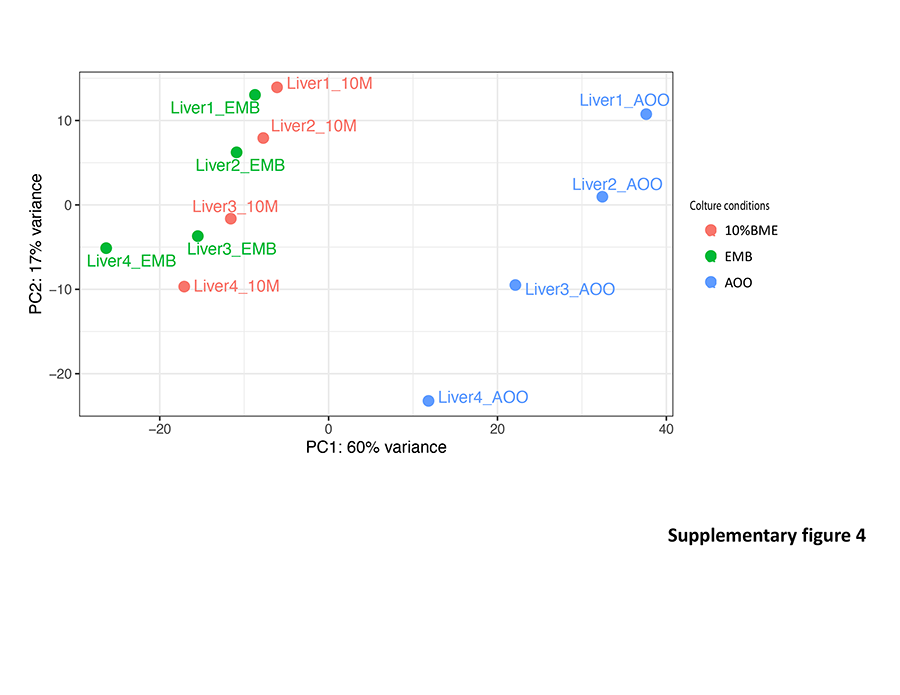

Supplement: Supplementary file 7 [file hc9-7-e0094-s004.tif]

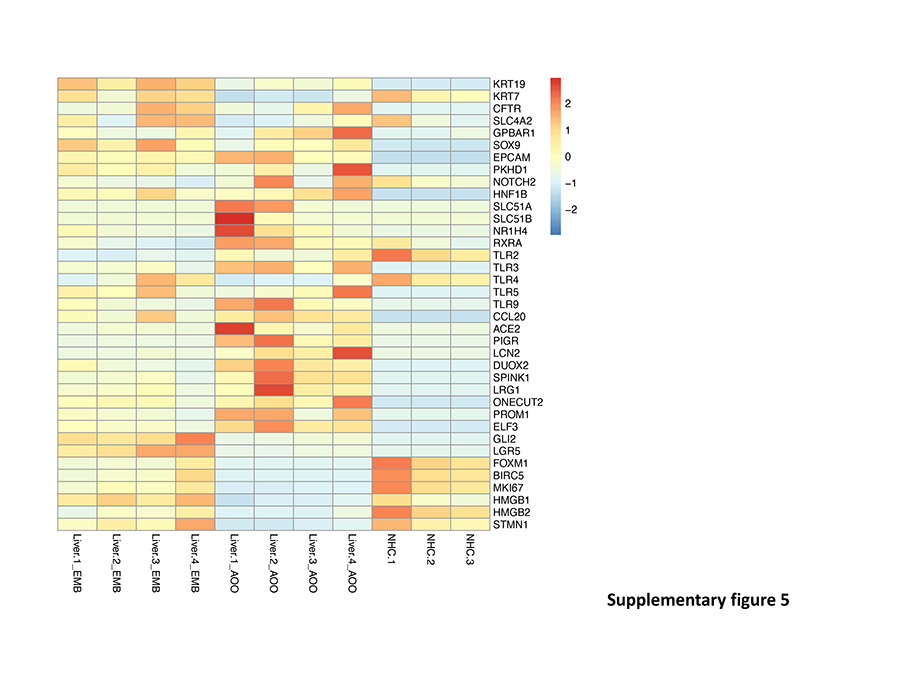

Supplement: Supplementary file 8 [file hc9-7-e0094-s005.tif]

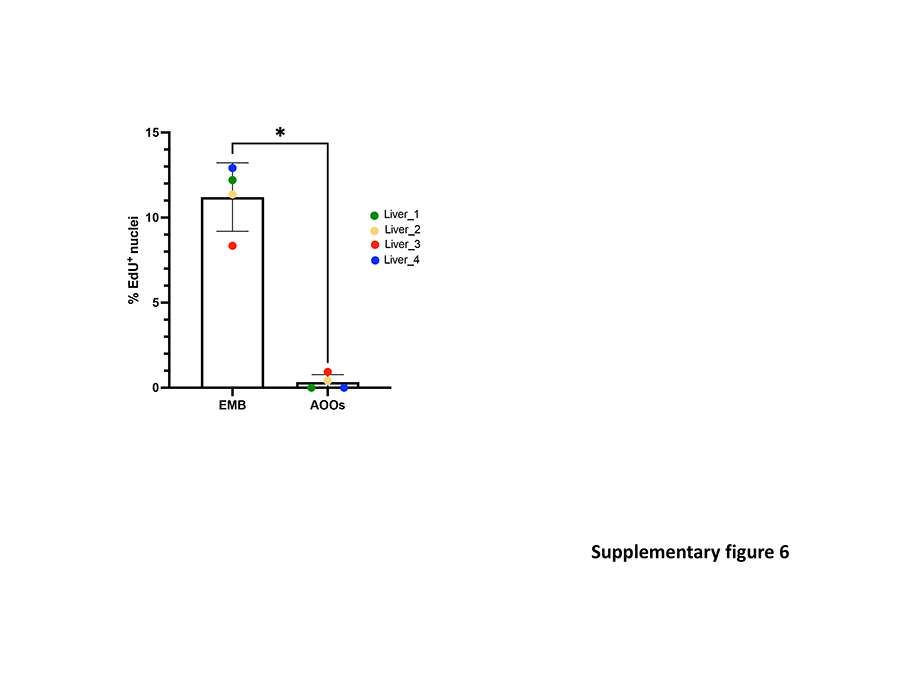

Supplement: Supplementary file 9 [file hc9-7-e0094-s006.tif]

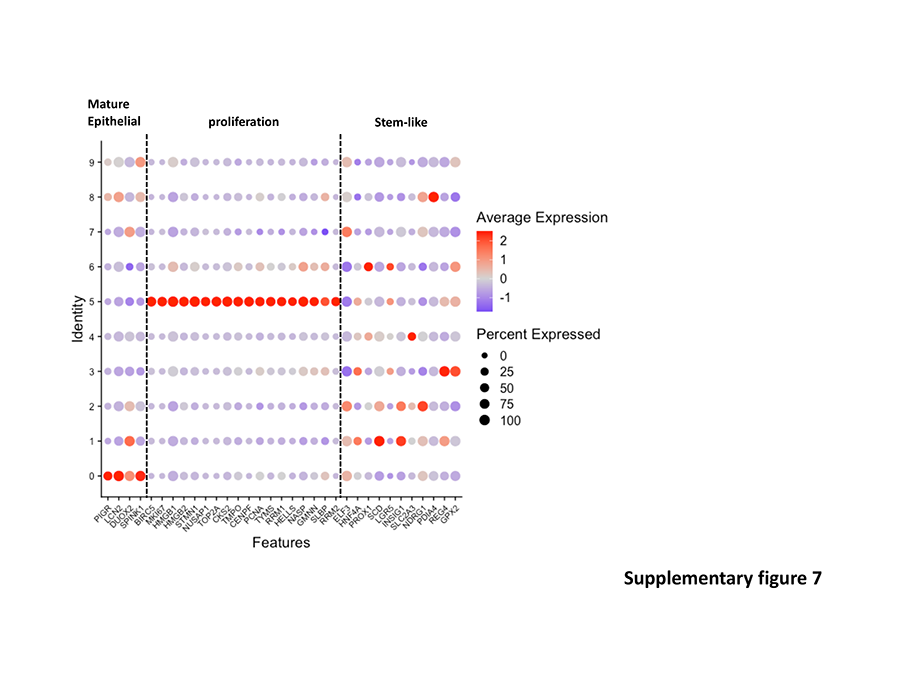

Supplement: Supplementary file 10 [file hc9-7-e0094-s007.tif]
