# Supplementary material for: Cell-matrix interactions control biliary organoid polarity, architecture, and differentiation
Source: Hepatol Commun. 2023 Mar 24;7(4):e0094. doi: 10.1097/HC9.0000000000000094 (PMC10503667; doi:10.1097/HC9.0000000000000094)
Supplement: Supplementary file 15 [file hc9-7-e0094-s012.docx]

Manuscript # HEP4-22-0733

**Supplementary material**

**Supplementary methods**

**Human biliary organoids generation and culture**

Briefly, liver tissues (about 1cm^3^) were kept in cold Advanced/DMEM-F12 until processing. Tissue samples were minced and digested in pre-warmed liver Digestion Solution *(Earle's Balanced Salt Solution (EBSS) with 2.5 mg/ml of Collagenase D and 0.1mg/ml DNase I)* for up to 90 minutes at 37°C and until the fragments were completely digested and single cells visible in the suspension. After washing with cold Wash Medium *(Dulbecco's Modified Eagle Medium (DMEM) supplemented with 1% Fetal Bovine Serum (FBS), 1% Penicillin-Streptomycin and 1% Sodium Pyruvate)* cells were passed through a 70 μm filter to remove debris and, the filtrated solution centrifugated at 300g for 5 minutes at 4°C. Cells were counted and resuspended in ice-cold Cultrex® Reduced Growth factors Basement Membrane Extract (BME) Type 2 (R&D Systems), and 50 μl/well domes with the mix of cell and BME suspension were allowed to polymerize in a 24-well plate at 37°C for 30 min. BME domes were overlaid with conditioned Isolation Medium *(Advanced DMEM/F12 supplemented with 30% Wnt3a conditional medium, 10% R-spondin conditional medium, 1% Penicillin-Streptomycin, 1% L-Glutamin, 2% B27 (Thermo Fisher Scientific), 10 mM nicotinamide (Sigma-Aldrich), 1 mM N-acetyl cysteine (Sigma-Aldrich), 1% N2 (Thermo Fisher Scientific),10 nM gastrin (Sigma-Aldrich), 50 ng/ml HGF (PeproTech), 50 ng/ml EGF (PeproTech), 5 μM transforming growth factor-β inhibitor (Tocris), 100 ng/ml fibroblast growth factor-10 (PeproTech, Germany), and 10 μM forskolin (Tocris)).* After 3 days of culture, medium was changed to Expansion Media (Isolation medium without Wnt3a) and replaced every 2 days.

Organoids embedded in BME were passaged every 7-10 days. Briefly, domes of BME type II Matrigel were mechanically disrupted by scraping and a suspension of cells in small clumps was obtained by pipetting in cold Basal Medium. Cells were spun down at 300g for 5 minutes at 4°C and either resuspended with BME and plated in BME domes or re-suspended in Isolation media containing 10%v/v of BME. Isolation media is used for the first three days in culture and then changed to Expansion media.

**Imaging of AOOs**

The switch in cell polarity in AOOs was confirmed by the change in organoid morphology under a transmitted light microscope Olympus CKX41. For live imaging, after removal of the matrix, organoids were labelled with CellMask Orange (Thermo Fisher Scientific) and transferred on the stage of a Bruker Opterra II swept-field confocal microscope (Billerica), at 37^o^C in a humidified atmosphere containing 5% CO_2_. Images were acquired at 5-mins intervals for 10-hrs and processed using Image J software.

**Transmission Electron Microscopy**

The liver organoids (EMB and AOOs) were fixed at room temperature in 2.5% glutaraldehyde and 2% paraformaldehyde in 0.1M cacodylate buffer (pH 7.4) for 1 hour, then post-fixed in 1% OsO_4_ in the same buffer at room temperature for 1 hour. After stained en bloc with 2% aqueous uranyl acetate for 30 min, tissue was dehydrated in a graded series of ethanol to 100% and embedded in EMbed 812 resin. Sample blocks were then polymerized in 60°C oven for 24 hr. 60 nm-thin sections (60 nm) were cut by a Leica UC7 ultramicrotome and post-stained with 2% uranyl acetate and lead citrate. The cell sections were examined with a FEI Tecnai transmission electron microscope at 80 kV of accelerating voltage, digital images were recorded with an Olympus Morada CCD camera and iTEM imaging software.

**Immunofluorescence**

BME-embedded and AOOs human biliary organoids were fixed in 4% paraformaldehyde in phosphate buffer solution (PBS) for 45 minutes and washed with IF buffer (PBS/BSA 0.1%/TritonX-100 0.2%/Tween20 0.1%). After permeabilization with PBS/TritonX-100 1%, unspecific binding sites were blocked with PBS/TritonX-100 1%/BSA 5% for 1 hour at RT. Cells were then incubated overnight at 4°C with specific primary antibodies (see supplementary table 1) and with the proper secondary antibody for 1 hour at RT. For laminin staining, an antigen retrieval step was performed before permeabilization with 10 mM citrate buffer/Tween20 0.05%, pH:6 for 20 minutes at 96-98 °C and secondary antibodies were incubated for 72 hours at 4°C. Nuclei were stained with TO-PRO™-3 Iodide (642/661) or DAPI. Confocal analysis was performed using a Zeiss LSM 710 Duo confocal microscope with a Plan-. Apochromat 20x/0.8. M27 objective.

**RNA Extraction and qRT-PCR**

RNA was isolated from organoids using RNeasy kit (QIAGEN) according to the manufacturer's instructions. cDNA was prepared from 1μg RNA using the TaqMan Reverse Transcription Kit (Thermo Fisher Scientific). The mRNA expression of specific genes was analyzed by quantitative PCR (qPCR) assay using commercially available specific FAM conjugated probes (Thermo Fisher Scientific) in combination with the Fast Start Universal Probe Master mix (Rox) (Roche Diagnostics, Indianapolis, IN) on an Applied Biosystems 7500 Real-Time PCR system. The expression level was normalized with the expression of the housekeeping gene GAPDH and analyzed using the ΔΔCt method. The genes analyzed are listed in supplementary table 1.

**Cholyl-lysyl-fluorescein (CLF) transport assay**

Cholyl-lysyl-fluorescein, CLF (Corning) a fluorescent bile salt derivative, was used to determine the bile acid transport activity of EMB and AOOs organoids. CellMask Orange (Thermo Fisher Scientific) plasma membrane stain was used on live organoids to label the plasma membrane following the manufacturer's instructions. After staining organoids were rinsed with 1x Hanks Balanced Salt Solution (Invitrogen) and incubated with 5μm CLF in HBSS for 30 minutes at 37°C. After the incubation period, the organoids were washed with HBSS and imaged live using a Zeiss LSM 710 Duo confocal microscope with a Plan-. Apochromat 20x/0.8. M27 objective.

**Epithelial barrier integrity**

Epithelial barrier integrity in AOOs organoids was determined by visualizing the paracellular passage of fluorescein isothiocyanate (FITC)-labeled 4-kDa dextran (Sigma-Aldrich). Briefly, AOOs were untreated or treated with glycochenodeoxycholic acid (GCDCA, 500 uM) for 24 hours. After treatment, organoids were pelleted and re-suspended in a solution of 4 kDa FITC-Dextran (2 mg/mL diluted in expansion medium) (Sigma). As a control for disrupted barrier integrity, AOOs were incubated with 2 mM EDTA. Paracellular diffusion of the dextran was live imaged using a Zeiss LSM 710 Duo confocal microscope with a Plan-. Apochromat 20x/0.8. M27 objective.

**Proliferation assay**

EdU staining to analyze cell proliferation was performed in EMB and AOOs (n=4 organoid lines) using the Click-iT EdU Alexa Fluor 488 kit (ThermoFisher Scientific). Organoids in both configuration (AOOs and EMB) were compared for each line at the same passage. Organoids were incubated with 5 μM EdU for 1 hr followed by fixation for 15 min with 3.7% formaldehyde. The Click-iT reaction cocktail was added according to manufacturer’s protocol and incubated for 30 min. Nuclear stain (Hoechst 33342, 10 μg/ml) was added for 20 min. Random images of organoids were collected with a Zeiss Axiovert epifluorescence microcope. Numbers of EdU positive cells were counted and % EdU positive cells versus nuclei were calculated.

**Bulk RNA-sequencing**

RNA was isolated from all organoid lines (n=4) cultured as indicated using RNeasy kit (QIAGEN) according to the manufacturer's instructions and submitted to the Yale Center for Genomic Analysis for library preparation and sequencing. For sequencing, sample concentrations were normalized to 1.2 nM and loaded onto an Illumina NovaSeq flow cell at a concentration that yields 25 million passing filter clusters per sample. Samples were sequenced using 100bp paired-end sequencing on an Illumina NovaSeq6000 according to Illumina protocols.

**Bulk RNA-sequencing computational analysis**

For bulk RNAseq, low quality reads were trimmed, and adaptor contamination were removed using Trim Galore (v0.5.0). Trimmed reads were mapped to the human reference genome (hg38) using HISAT2 (v2.1.0). Gene expression levels were quantified using StringTie (v1.3.3b) with gene models (v27) from the GENCODE project. Differentially expressed genes (DEGs) were identified using DESeq2 (v 1.22.1). The DEGs satisfying the conditions of the fold change cut-off 2 and a One-way analysis of variance with a padj value < 0.05 for all the genes probed in the array. The lists of DEGs from the EMB, 10% BME and AOOs organoids were analyzed for gene ontology enrichment using the EnrichR web site <https://maayanlab.cloud/Enrichr/>.

**scRNA-seq Library preparation, sequencing, and raw data processing**

For 10x Genomics, single-cell RNA sequencing, 10,000 cells were loaded onto 10x Genomics Single Cell 3′ Chips along with the master mix as per the manufacturer’s protocol for the Chromium Single Cell 3′ Library to generate single cell gel beads in emulsion (GEMs, version 3.1 chemistry), followed by cell lysis and barcoded reverse transcription of RNA in the droplets. The resulting libraries were sequenced on a NovaSeq6000. Raw base calls were demultiplexed and converted to fastq files using Cell Ranger mkfastq program (bcl2fastq 2.19/Cell Ranger 3.0). Sequencing data were first preprocessed through the Cell Ranger pipeline (10x Genomics, Cell Ranger count v2) with default parameters.

**Bioinformatics analysis of scRNA-seq data**

Data were analyzed in Seurat V3 using SCTransform normalization. Genes detected in at least 10 cells were included. Cells that expressed less than 200 and more than 5000 genes or had high mitochondrial genome transcript percentage (>15%) were excluded. Percent mitochondrial reads was regressed out of clustering/aggregation. Unsupervised clustering and differential gene expression analyses were performed in the *Seurat*R package v2.3.0(16). After clustering of all cells at resolution 0.8, clusters were identified by canonical markers (see Supplementary table 3). All heatmaps, UMAP visualizations, and feature plots were produced using *Seurat*functions in conjunction with the *ggplot2.* Differential gene expression analysis was conducted in *Seurat*to assess significance, retaining only those genes with a log-fold change of at least 0.25 and expression in at least 25% of cells in the cluster under comparison.

**Antibody treatments**

EMB and 10%BME biliary organoids (n=4 organoid lines) were treated with an anti-β1 integrin-neutralizing antibody clone AIIB2 (Developmental Studies Hybridoma Bank) and compared using bright field microscopy to untreated ones from day 1 to 4. *See Figure 5 legend for imaging analysis.*

**Bacteria culture and preparation**

*E. gallinarum* and *E. faecalis* (ATCC 51299) were grown in anaerobic conditions and GAM broth for 4-hrs until log-phase. Bacteria cultures were pelleted, washed in PBS twice, and labeled with 10uM carboxyfluorescein succinimidyl ester (CSFE)(Millipore) in PBS at room temperature for 1-hr. Bacteria culture was washed three times in 50mL of PBS and resuspended in expansion media without antibiotics to a 10^6^ CFU per mL. Bacteria were added to organoids culture, producing a final concentration of 10^5^ CFU per mL organoid culture.

**Cytokine array**

EMB and AOOs (n=4 organoid lines) were exposed to heat-inactivated Enterococcus spp (E. gallinarum liver, E. gallinarum faecalis and E. faecalis) for 6 hours and the supernatant collected to measure the secretion of the chemokine and cytokine by Proteome Profiler Human Cytokine Array Kit (R&D Systems), according to the manufacturer's instructions. Arrays were analyzed using HLImage++, Western Vision Software, Salt Lake City, UT.

**Supplementary figure and video legends**

**Supplementary figure 1. EMB and BME 10% organoids have a similar morphology.** Representative brightfield images showing the similar morphology between organoids cultured embedded in BME (EMB) or in suspension in a 10% BME/medium.

**Supplementary figure 2. EM images showing the intracellular ultrastructure of EMB and AOOs organoids.** Nu, nucleus; Tj, tight junction, M, mitochondria; scale bars are as indicated.

**Supplementary figure 3. Apical-out organoids express biliary markers and down-regulated LGR5.** Bar graphs show qRT-PCR gene expression levels (normalized to GAPDH) of known biliary markers, bile acid transporters and LGR5 in AOOs and EMB derived from 4 livers.

**Supplementary figure 4. PCA plot of variance for the top 200 differentially expressed genes among EMB, 10% BME and AOOs generated from 4 livers.**

**Supplementary figure 5. Gene expression of biliary/epithelial markers is higher in AOOs as compared to EMB and normal human cholangiocytes in culture.** Gene counts from bulk-RNAseq in AOOs and EMB were compared with a publicly available data set from NHC cells (GSE146899). Heatmap showing the differential expression comparison of selected genes related to biliary differentiation markers, pathogens defence related genes, mature epithelium markers, stemness and proliferation markers.

**Supplementary figure 6. Proliferation is significantly decreased in AOOs.** Bar graph shows the average percentage of EdU positive cells versus the total nuclei in n=10 random fields for each organoid line (n=4 lines) in the configuration EMB and AOOs. Proliferation is significantly decreased in AOOs; *p<0.05.

**Supplementary figure 7. Distribution of different cholangiocyte populations among the cell clusters identified by scRNA-Seq.** The dot plot shows the expression of selected genes used to characterize populations of cholangiocytes along the different clusters. The size of the circle identifies the percentage of cells expressing each gene while the color indicates the average expression level.

**Supplementary video 1. The change of polarity in the absence of ECM is rapid and involves a mechanism of folding and eversion.** EMB organoids were removed from the ECM, labeled with CellMask Orange and live recorded for 10 hours.

**Supplementary video 2. Presence of laminin in the internal cavity of AOOs in contact with the basolateral membrane.** Confocal Z-stack series in fixed AOOs, showing the expression of laminin in the inside cavity of AOOs, in contact with the basolateral membrane of the organoid marked by β catenin staining.

**Supplementary video 3. Live imaging of bacteria-organoids in co-culture.** *E. faecalis* bacteria labelled with CSFE were co-cultured with AOOs labelled with CellMask Orange and live recorded for 8 hours. The video shows that bacteria get in physical contact with the apical surface of the organoid.

**Supplementary file 1. Tables with differentially expressed genes (Bulk) of EMB versus AOOs, EMB versus 10% BME and AOOs versus 10% BME with organoids originated from n=4 individual liver tissues.**

**Supplementary file 2. Tables with gene counts from organoids EMB, AOOs and NHC cells RNA-seq.**

**Supplementary file 3. Tables with GO analysis of biological processes derived from the comparison of AOOs and EMB organoids.**

**Supplementary file 4. Tables with differentially expressed genes (scRNA-seq) in unsupervised clusters and cholangiocyte populations.**

| **REAGENT** | **SOURCE** | **IDENTIFIES** |
| --- | --- | --- |
| **PCR probes** | | |
| CFTR | Thermo Fisher Scientific | Hs00357011_m1 |
| SLC4a2 (AE2) | Thermo Fisher Scientific | Hs01586776_m1 |
| Keratin 19 | Thermo Fisher Scientific | Hs01051611_gH |
| SOX9 | Thermo Fisher Scientific | Hs01001343_g1 |
| LGR5 | Thermo Fisher Scientific | Hs00173664_m1 |
| SCTR | Thermo Fisher Scientific | Hs01085380_m1 |
| SLC10A2 (ASBT) | Thermo Fisher Scientific | Hs0100557_m1 |
| SLC51A (OSTα) | Thermo Fisher Scientific | Hs00380895_m1 |
| SLC51B (OSTβ) | Thermo Fisher Scientific | Hs01057182_m1 |
| MRP3 | Thermo Fisher Scientific | Hs00978473_m1 |
|  |  |  |
| **Antibodies** |  |  |
| Mouse anti-β-Catenin | BD Biosciences | Cat# 610153 |
| Rabbit anti-β-Catenin | Cell Signaling | Cat# 9562 |
| Mouse anti-CFTR | R&D Systems | Cat# MAB25031 |
| Rabbit anti-ASBT | Dr. Paul Dawson |  |
| Rabbit anti-ZO-1 | Thermo Fisher Scientific | Cat# 61-7300 |
| Rabbit anti-Laminin | Sigma-Aldrich | Cat# L9393 |
| Mouse anti acetylated alpha-tubulin | Sigma-Aldrich | Cat# T6793 |
|  |  |  |
| **Fluorescent dyes** |  |  |
|  |  |  |
| TO-PRO-3 Iodide | Thermo Fisher Scientific | T3605 |
| CellMask^TM^ Orange | Thermo Fisher Scientific | C10045 |

**Supplementary table 1.**
